# Cannabis Therapeutics and the Future of Neurology

**DOI:** 10.3389/fnint.2018.00051

**Published:** 2018-10-18

**Authors:** Ethan B. Russo

**Affiliations:** International Cannabis and Cannabinoids Institute (ICCI), Prague, Czechia

**Keywords:** cannabis, pain, brain tumor, epilepsy, Alzheimer disease, Parkinson disease, traumatic brain injury, microbiome

## Abstract

Neurological therapeutics have been hampered by its inability to advance beyond symptomatic treatment of neurodegenerative disorders into the realm of actual palliation, arrest or reversal of the attendant pathological processes. While cannabis-based medicines have demonstrated safety, efficacy and consistency sufficient for regulatory approval in spasticity in multiple sclerosis (MS), and in Dravet and Lennox-Gastaut Syndromes (LGS), many therapeutic challenges remain. This review will examine the intriguing promise that recent discoveries regarding cannabis-based medicines offer to neurological therapeutics by incorporating the neutral phytocannabinoids tetrahydrocannabinol (THC), cannabidiol (CBD), their acidic precursors, tetrahydrocannabinolic acid (THCA) and cannabidiolic acid (CBDA), and cannabis terpenoids in the putative treatment of five syndromes, currently labeled recalcitrant to therapeutic success, and wherein improved pharmacological intervention is required: intractable epilepsy, brain tumors, Parkinson disease (PD), Alzheimer disease (AD) and traumatic brain injury (TBI)/chronic traumatic encephalopathy (CTE). Current basic science and clinical investigations support the safety and efficacy of such interventions in treatment of these currently intractable conditions, that in some cases share pathological processes, and the plausibility of interventions that harness endocannabinoid mechanisms, whether mediated via direct activity on CB_1_ and CB_2_ (tetrahydrocannabinol, THC, caryophyllene), peroxisome proliferator-activated receptor-gamma (PPARγ; THCA), 5-HT_1A_ (CBD, CBDA) or even nutritional approaches utilizing prebiotics and probiotics. The inherent polypharmaceutical properties of cannabis botanicals offer distinct advantages over the current single-target pharmaceutical model and portend to revolutionize neurological treatment into a new reality of effective interventional and even preventative treatment.

## Introduction

Cannabis burst across the Western medicine horizon after its introduction by William O’Shaughnessy in 1838 (O’Shaughnessy, 1838–1840; Russo, 2017b), who described remarkable successes in treating epilepsy, rheumatic pains, and even universally fatal tetanus with the “new” drug. Cannabis, or “Indian hemp,” was rapidly adopted by European physicians noting benefits on migraine by Clendinning in England (Clendinning, 1843; Russo, 2001) and neuropathic pain, including trigeminal neuralgia by Donovan in Ireland (Donovan, 1845; Russo, 2017b). These developments did not escape notice of the giants of neurology on both sides of the Atlantic, who similarly adopted its use in these indications: Silas Weir Mitchell, Seguin, Gowers and Osler (Mitchell, 1874; Seguin, 1877; Gowers, 1888; Osler and McCrae, 1915). While medicinal cannabis suffered a period of obscurity and quiescence, mainly attributable to quality control issues and political barriers, modern data on migraine (Russo, 2004, 2016b; Rhyne et al., 2016) and neuropathic pain, whether central or peripheral support its common application by affected patients (Rog et al., 2005; Nurmikko et al., 2007; Russo and Hohmann, 2013; Serpell et al., 2014), additionally supported by the National Academies of Science, Engineering and Medicine (National Academies of Sciences Engineering and Medicine (U.S.). Committee on the Health Effects of Marijuana: An Evidence Review and Research Agenda, 2017).

It has been noted for some time that muscle tone on the central level is mediated by the endocannabinoid system (Baker et al., 2003), but some additional years were necessary to bring this “aspirin of the 21st century” through Phase I–III Randomized Clinical Trials (RCTs; Novotna et al., 2011) and post-marketing assessment to demonstrate its safety, efficacy and consistency (Rekand, 2014; Fife et al., 2015; Maccarrone et al., 2017). That preparation, nabiximols (US Adopted Name; Sativex^®^) has currently attained regulatory approval in 30 countries for spasticity associated with multiple sclerosis (MS), and in Canada for central neuropathic pain in MS (Rog et al., 2005), and for opioid-resistant cancer pain (Johnson et al., 2010). Recent surveys find usage rates for cannabis of 20%–60% among MS patients (Rudroff and Honce, 2017). An earlier attempt to demonstrate neuroprotection in head trauma after intravenous administration of single doses of the non-intoxicating cannabinoid analog, dexanabinol, failed (Maas et al., 2006), but hope remains for other preparations in stroke and other brain insults (Latorre and Schmidt, 2015; Russo, 2015; Pacher et al., 2018). Table 1 summarizes the current status of cannabis-based drugs in neurological conditions not discussed at length herein, including sleep disturbance (Russo et al., 2007; Babson et al., 2017), glaucoma (Merritt et al., 1980), lower urinary tract symptoms (LUTS; Brady et al., 2004; Kavia et al., 2010), social anxiety (Bergamaschi et al., 2011), Tourette syndrome (Müller-Vahl et al., 2002, 2003) and schizophrenia (Leweke et al., 2012; McGuire et al., 2018). This Perspective article will rather focus on several neurological syndromes that overlap in their pathophysiology or have yet to receive concerted attention in clinical trials of cannabis-based medicines.

**Table 1:** Neurological conditions for which cannabis-based treatments have been employed (revised, reformatted and supplemented from MacCallum and Russo, 2018).

Condition	Preparation	Level of evidence	Type of evidence
Multiple sclerosis (MS) spasticity	Nabiximols	Conclusive	Phase III RCTs, Regulatory approval
Epilepsy (Dravet and Lennox-Gastaut syndromes)	Cannabidiol (Epidiolex^®^)	Conclusive	Phase III RCTs, Regulatory approval
Chronic pain	THC, nabiximols	Substantial	Phase II RCTs
Schizophrenia, positive and negative symptoms	CBD	Substantial	Phase II RCTs
Sleep disturbance secondary to neurological symptoms	THC, nabilone, nabiximols	Moderate	Phase II–III RCTs
Glaucoma	THC, cannabis	Moderate	Phase II RCTs
Lower urinary tract symptoms (LUTS) in MS	Nabiximols	Moderate	Phase II RCTs
Tourette syndrome	THC, cannabis	Moderate	Phase II RCTs, observational studies
Dementia with agitation	THC, cannabis	Limited	Observational studies
Parkinson disease symptoms	THC, CBD, cannabis	Limited	Observational studies
Post-traumatic stress disorder	Cannabis	Limited	Observational studies
Social anxiety	CBD	Limited	Phase II RCT, observational studies

This author has previously addressed the pathophysiology of migraine (Sarchielli et al., 2007), post-traumatic stress (Hill et al., 2013), Parkinson disease (PD; Pisani et al., 2005) and other conditions as putative clinical endocannabinoid deficiency disorders wherein disturbances in endocannabinoid tone have been demonstrated objectively (Russo, 2004, 2016b).

Various synthetic fatty acid amidohydrolase (FAAH) inhibitors have been investigated for neurological therapeutics (Nozaki et al., 2015), but none have advanced to Phase III clinical trials. This is a mechanism of action seemingly shared with cannabidiol (Bisogno et al., 2001).

## Cannabis and Epilepsy

**Figure 1:**
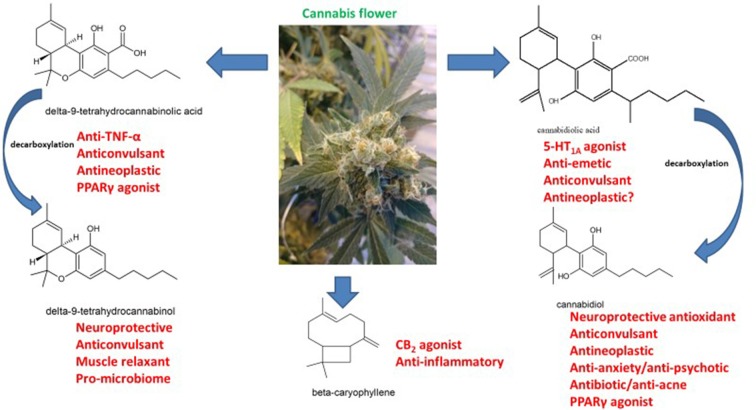
The pharmacology of phytocannabinoids pertinent to treatment of neurodegenerative disorders (molecular structures drawn by ER with ACD/ChemSketch 2015.2.5).

After elucidation of phytocannabinoid structures in the 1960s, their pharmacology was slowly revealed (reviewed by Cascio and Pertwee, 2014; Pertwee and Cascio, 2014; Russo and Marcu, 2017; Figure 1). Various components were tested for anticonvulsant activities with findings of ED_50_ in mice of 80 mg/kg for tetrahydrocannabinol (THC), 120 mg/kg for cannabidiol (CBD) and 200 mg/kg for tetrahydrocannabinolic acid A (THCA-A), the carboxylic acid precursor to THC found in raw cannabis flowers (Karler and Turkanis, 1979). Although dose-response was tested, it is unclear that very low doses were assessed and given the biphasic tendencies of cannabinoids, it is possible that positive lower dose effects may have remained unnoticed. CBD was considered an excellent candidate for development based on its lack of untoward psychoactive sequelae. However, little work was done until a series of small human trials in Brazil in following decades (reviewed by Russo, 2017a).

Subsequent investigation demonstrated that seizure threshold is mediated by the endocannabinoid system (Wallace et al., 2003), and that THC produced a 100% reduction in seizures, whereas phenobarbital and diphenylhydantoin did not. Additionally, animal studies demonstrated both acute increases in endocannabinoid production and a long-term up-regulation of CB_1_ production as apparent compensatory effects counteracting glutamate excitotoxicity, and that anticonvulsant effect was present at sub-sedating levels.

Sporadic case reports of successful utilization of THC in seizures associated with severe neurological conditions in children in Germany followed (Lorenz, 2004; Gottschling, 2011), but the prime focus returned to CBD due to strong anticonvulsant results in laboratory investigation (Jones et al., 2010), which led directly to a pharmaceutical development program. The lay public quickly became aware of these developments, with promotion of the concept by Project CBD^1^ and publicity associated with the case of Charlotte Figi and significant improvement in seizures associated with Dravet syndrome, as portrayed on the *Weeds* documentary on Cable News Network (Maa and Figi, 2014). Positive survey results (Porter and Jacobson, 2013) were tempered, however, by studies suggesting strong ascertainment bias in parental reporting of seizure frequency: response rate for families moving to the state of Colorado for cannabidiol treatment was 47% vs. only 22% for those already living there, and were three-fold higher for those reporting >50% response (Press et al., 2015). More careful observational studies with a standardized cannabidiol oral extract with THC removed (Epidiolex^®^) provided more compelling results (Devinsky et al., 2016) with a 55% median reduction in seizures in Dravet and Lennox-Gastaut Syndrome (LGS) patients at high dose. Subsequent Phase III results in Dravet syndrome at CBD 20 mg/kg/d showed strong statistical significance in seizure frequency and Caregiver Global Impression of Change (Devinsky et al., 2017). More recent studies have bolstered evidence for safety and efficacy of the preparation in both conditions (Devinsky et al., 2018; Thiele et al., 2018). As a result, it received US Food and Drug Administration approval in June 2018.

Interestingly, extensive observations from other practitioners (Russo et al., 2015) seemed to indicate similar therapeutic successes with much lower doses of CBD when utilized in cannabis-based preparations with small concomitant amounts of THC, THCA and linalool, a terpenoid component of cannabis (Russo, 2017a; Sulak et al., 2017; Pamplona et al., 2018). Selective cannabis breeding via Mendelian techniques raises the possibility of producing chemovars with multiple anticonvulsant components that may produce synergistic benefits (Lewis et al., 2018). THCA is an intriguing issue, in that there is debate about whether it harbors CB_1_ activity, or rather is due to spontaneous decarboxylation to THC (McPartland et al., 2017; Figure 1). Cannabidiolic acid (CBDA) was also recently reported to demonstrate anticonvulsant activity (Bonni Goldstein, personal communication), possibly attributable to its serotonergic activity (Bagdy et al., 2007), in that CBDA demonstrates 100-fold greater affinity for the 5-HT_1A_ receptor (Bolognini et al., 2013) as compared to CBD (Russo et al., 2005).

## Cannabis and Brain Tumors

Strong scientific evidence of cytotoxic benefit of phytocannabinoids has been available since 1975 (Munson et al., 1975) and highlighted three decades later (Ligresti et al., 2006), but the historical record suggests ancient use by Egyptian Copts (THC and/or THCA; Reymond, 1976; Russo, 2007) with similar claims by Renaissance herbalists in Europe (CBD and/or CBDA; Russo, 2007). Brain tumors are the subject of an excellent current review (Dumitru et al., 2018). To summarize available research, specific pro-apoptotic activity of THC in C6 glioma was reported (Sánchez et al., 1998), and shrinkage of *in situ* human glioma cell line tumors was observed with CBD (Massi et al., 2004). Intra-tumoral THC administration in glioblastoma multiforme (GBM) produced slight life prolongation over expectations in nine human patients (Guzmán et al., 2006). Case reports from Canada documented total regression of residua in two pilocytic astrocytomata in children after smoked cannabis (Foroughi et al., 2011). Careful laboratory analysis has established synergistic benefits of combinations of THC, CBD and standard chemotherapy with temozolomide on glioma (Torres et al., 2011). Clinical application of the concept has been reported online in a Phase II randomized controlled trial (RCT) of 21 patients with recurrent GBM on temozolomide plus nabiximols up to 12 sprays per day (32.4 mg THC plus 30 mg CBD plus terpenoids) vs. placebo with an 83% 1-year survival vs. 53% in controls (*p* = 0.042) and survival exceeding 550 days vs. 369 for controls, and only two withdrawals in each group due to adverse events (AEs)^2^.

Such encouraging results are supplemented by a recent report that THCA is a peroxisome proliferator-activated receptor-gamma (PPARγ) agonist (IC_50_ = 470 nM, K_i_ = 209 nM) > CBGA (517.7 nM) and ≫ than CBDA, CBD or THC (Nadal et al., 2017). THCA improved neuronal viability in an animal model of Huntington disease (HD), and decreased striatal neurodegeneration (blocked by PPARγ antagonist), and it was suggested as a therapeutic agent in HD. This finding, however, has much larger implications and could explain claims of therapeutic efficacy in epilepsy noted above (Sun et al., 2008), as well tumors, and perhaps even in major depression (Colle et al., 2017a,b). In contrast to other neutral cannabinoids and terpenoids, THCA is reported not to cross the blood-brain barrier (BBB), but if true, that hindrance may not be applicable in the context of chronic epilepsy (Oby and Janigro, 2006), or in brain tumors wherein that barrier is compromised.

As reviewed (Elrod and Sun, 2008), PPARs are ligand-binding transcription factors on nuclear membranes that affect adipogenesis, apoptosis and many other functions. PPARγ stimulation may kill cancer cells without toxicity to normal cells, such as astrocytes, and their effects are additive with other cytotoxic agents. Butyrate and capsaicin may be natural ligands. PPARγ has been identified in many cancers including those affecting the brain, where it regulates target gene transcription (Shen et al., 2016), and its activation inhibits tumor cell growth. These authors suggested that PPARγ agonist may prove useful in treating brain tumors, and may extend as well to “benign” lesions, such as meningioma, wherein pioglitazone demonstrated activity (Gehring et al., 2011; Shen et al., 2016).

Thus, a Type II cannabis preparation, with equal THC and CBD concentration, combining THC, CBD, THCA and even CBDA along with cytotoxic terpenoids such as limonene may prove extremely useful in cancer treatment (Lewis et al., 2018).

## Cannabis and Parkinson Disease (PD)

As early as 1888, Gowers noted benefits of “Indian hemp” on a parkinsonian syndrome (Gowers, 1888; Russo, 2007). Because of the density of cannabinoid receptors in basal ganglia, PD has been an area of active research, but with mixed results therapeutically. An oral THC:CBD extract showed no significant benefits on dyskinesia or other signs in 17 patients (Carroll et al., 2004), but CBD was helpful in five PD patients with psychosis (Zuardi et al., 2009) and 21 patients with more general symptoms (Chagas et al., 2014b) and more specifically on rapid eye movement sleep disorder in four patients (Chagas et al., 2014a). An observational study showed 22/28 patients tolerated smoked cannabis (presumably THC-predominant) and showed acute benefits on tremor, rigidity and bradykinesia (Lotan et al., 2014). Five of nine patients using cannabis reported great improvement, particularly on mood and sleep (Finseth et al., 2015).

A carefully crafted survey of 339 Czech patients using oral cannabis leaves reported significant alleviations of multiple symptoms (Venderová et al., 2004), particularly those using the treatment for three or more months, with improvement in general function (*p* < 0.001), resting tremor (*p* < 0.01), bradykinesia (*p* < 0.01), and rigidity (*p* < 0.01) with few side effects.

Whereas PD is commonly attributed to cell loss in the substantia nigra, with chronicity, widespread pathology is the norm. In common with Alzheimer disease (AD), tau proteins that regulate microtubule assembly, cytoskeletal integrity and axonal transport in neurons develop neurofibrillary tangles (Lei et al., 2010). Interestingly, nabiximols reduced such tangles in parkin-null human tau-expressing mice with improvement in dopamine metabolism, glial function and oxidative stress, as well as reducing anxiety and self-injury (Casarejos et al., 2013).

## Cannabis and Alzheimer Disease (AD)

Recent reviews (Aso and Ferrer, 2014; Ahmed et al., 2015) have nicely summarized the pathophysiology of AD: a neurodegenerative disease with senile plaques formed of fibrillar β-amyloid (Aβ) from cleavage of the Aβ precursor protein (APP) by β- and γ-secretases and by presence of neurofibrillary tangles composed of hyper-phosphorylated and nitrated tau protein. The latter precedes Aβ deposition in sporadic cases. Once the process begins, deterioration is inexorable. Additional pathology includes functional mitochondrial defects, increased reactive oxygen species (ROS) and reactive nitrogen species (RNS), and failure of enzymes involved in energy production that, in turn, produces nerve cell exhaustion. Eventually, synapses and dendritic branching fail, with consequent progressive neuronal wastage. Dementia and cognitive decline develop, and no treatment arrests the process. Intervention must begin at an early preclinical stage to have any hope of success. Endocannabinoid function modulates the primary pathological processes of AD during the silent phase of neurodegeneration: protein misfolding, neuroinflammation, excitotoxicity, mitochondrial dysfunction and oxidative stress. CB_2_ levels increase in AD especially in microglia around senile plaques, and its stimulation stimulates Aβ removal by macrophages.

The epidemiology of AD is fascinating (Mayeux and Stern, 2012). North America and Western Europe have highest rates (6.4% and 5.4% at age 60), then Latin America (4.9%), and China (4%; ascertainment bias vs. mirroring economic development and Western diet?). Prevalence is lower for Africans in homelands, as opposed to higher rates in the Western European and American diaspora. Head trauma increases Aβ deposition and neuronal tau expression, and diabetes, obesity, trans-fats and head trauma all increase AD risk. Mediterranean diet (increased monounsaturated olive oil, and omega-3 from fish), education and physical activity reduce it.

No current pharmacotherapy is approved for agitation in AD. Commonly used anti-psychotics, antidepressants, anxiolytics and hypnotics are often associated with increased mortality in demented patients (Kales et al., 2007), with an FDA “Black Box Warning.” Four acetylcholinesterase inhibitors are approved in the USA to improve memory: galantamine, donepezil, tacrine and rivastigmine. None show strong evidence of efficacy and are of limited benefit on a temporary basis. Various NMDA receptor antagonists in development have proven largely ineffective on disease progression or have proven toxic. In contrast, treatment with cannabinoids appears both more promising and benign. As demonstrated in 1998 (Hampson et al., 1998), and the subject of USA patent US09674028, CBD is a neuroprotective antioxidant, more potent than ascorbate or tocopherol, that works on the same NMDA target without attendant toxicity. Subsequently (Iuvone et al., 2004), CBD inhibited Aβ plaque formation, prevented ROS production and peroxidation of lipids in PC12 cells exposed to Aβ, limited neuronal apoptosis from caspase 3 reduction, and counteracted increases in intracellular Ca^++^ from Aβ. In an *in vivo* model (Esposito et al., 2006), CBD was anti-inflammatory via reduction in inducible nitric oxide synthase (iNOS) and IL-1β expression and release. It also inhibited tau protein hyper-phosphorylation in Aβ-stimulated PC12 neurons. Subsequently, it was shown that CBD’s MOA seemed to be selectively mediated via PPARγ (Esposito et al., 2011): dose dependently antagonizing pro-inflammatory NO, tumor necrosis factor-alpha (TNF-α), and IL-1β. That effect was blocked by GW9662 (PPARγ antagonist), reducing reactive gliosis via selective PPARγ-related NFκB inhibition. Both AEA and CBD promoted neurogenesis after Aβ exposure.

In addition to its neuroprotective antioxidant effects (Iuvone et al., 2004), THC competitively inhibited acetylcholinesterase, increasing levels, and prevented Aβ aggregation via binding to the enzyme in a critical region affecting amyloid production (Eubanks et al., 2006).

On the clinical side, various trials of THC in AD have produced positive results. In 1997 (Volicer et al., 1997), in 15 institutionalized dementia patients refusing nutrition, an RCT 6-week crossover trial of THC (Marinol^®^) 2.5 mg twice daily led to increased body-mass index (BMI), with decreased Cohen-Mansfield Agitation Inventory (CMAI) scores, improved negative affect scores, and a notable carry-over effect when THC was administered first. In 2006 (Walther et al., 2006), an open-label 2-week study of five AD and one vascular dementia patient taking THC 2.5 mg at 19:00 h showed benefit noted on nocturnal motor activity, agitation, appetite, and irritability with no AEs. A 2015 study (van den Elsen et al., 2015) failed, however: an RCT in 50 demented patients with neuropsychiatric symptoms received 1.5 mg THC vs. placebo thrice daily for 3 weeks with no benefit noted to THC. A total lack of AEs indicated to the even the authors that the administered dosage was inadequate and that higher doses might be required.

Initial trials of herbal cannabis for AD have begun sporadically, with a more focused effort in a California nursing home (Hergenrather, 2017). Patients were treated with a variety of preparations: THC-predominant (2.5–30 mg/dose), CBD predominant, and THCA, mainly in tinctures and confections. Marked benefit was reported on neuroleptic drug sparing, decreased agitation, increased appetite, aggression, sleep quality, objective mood, nursing care demands, self-mutilation and pain control.

Based on its pharmacology (Russo and Marcu, 2017), cannabis components may provide myriad benefits on target symptoms in this complex disorder:

- Agitation: THC, CBD, linalool
- Anxiety: CBD, THC (low dose), linalool
- Psychosis: CBD
- Insomnia/Restlessness: THC, linalool
- Anorexia: THC
- Aggression: THC, CBD, linalool
- Depression: THC, limonene, CBD
- Pain: THC, CBD
- Memory: alpha-pinene (Russo, 2011; Russo and Marcu, 2017) + THC
- Neuroprotection: CBD, THC
- Reduced Aβ plaque formation: THC, CBD, THCA

Thus, an extract of a Type II chemovar of cannabis (THC/CBD) with a sufficient pinene fraction would seem to be an excellent candidate for clinical trials (Lewis et al., 2018).

## Cannabis and Traumatic Brain Injury (TBI)/Chronic Traumatic Encephalopathy (CTE)

The neuroprotective antioxidant effects of the cannabinoids (Hampson et al., 1998) are particularly relevant in their ability to counteract “glutamate excitoxicity,” which leads to neuronal demise after traumatic brain injury (TBI). Anecdotally, cannabis, particularly chemovars combining THC and CBD, have been extremely helpful in treatment of chronic traumatic encephalopathy (CTE) symptoms: headache, nausea, insomnia, dizziness, agitation, substance abuse, and psychotic symptoms. CTE, previously known as *dementia pugilistica*, or “punch-drunk syndrome” has garnered a great deal of attention due to its apparent frequency among long-term players of American football but including victims of repetitive head injury from causes as diverse as other contact sports, warfare and even “heading” in soccer. A recent study (Mez et al., 2017) showed 87% of autopsied American football players demonstrated CTE with tau aggregates in neurons and astrocytes, neurofibrillary tangles in superficial cortical layers and hippocampus, α-synuclein and Aβ deposition. Microglia were present early in the course, whose premonitory symptoms include dementia, personality change, rage, and attention problems. Ninety-six percent demonstrated a degenerative course. Heretofore, this has been considered a post-mortem pathological diagnosis, but two current studies support the ability for pre-mortem identification. CCL11 protein is a chemokine associated with cognitive decline and enhances microglial production of ROS and excitotoxic cell death. CSF examination in CTE patients were elevated compared to controls and AD patients (*p* = 0.028), and correlated to years of football played (*p* = 0.04; Cherry et al., 2017), indicating CCL11 may be a premortem biomarker for the syndrome. Additionally, PET imaging binding levels in a CTE patient before death correlated with postmortem tau deposition (*p* = 0.02). The greatest tau concentration was observed in parasagittal and paraventricular cortical and brainstem areas (Omalu et al., 2018), allowing pre-mortem diagnosis and distinction from AD. Neuroprotective benefits of phytocannabinoids, particularly CBD, further outlined below, provide support for trials of these agents in post-traumatic syndrome and CTE prevention.

## Human Nutrition, Cannabis, the ECS, “Acne of the Brain” and the “Gut-Skin-Brain Axis”

Human gut harbors 100 trillion micro-organisms at a concentration of 10^12^ bacteria/ml, and exceeding the human genome 100-fold (Musso et al., 2010). This is termed the microbiome. Obese humans have lower Bacteroidetes and higher Firmicutes counts. Recent review (Clarke et al., 2012; Russo, 2016b) supports the efficacy of probiotics (supplemental beneficial gut lactic acid bacteria) in treating irritable bowel syndrome without AEs. Microbiota regulate 5-HT_1A_, BDNF and NMDA expression (Sampson et al., 2016), and experimental transplantation of the microbiome of Parkinson patients to mice was demonstrated to increase their motor deficits, supporting the finding of a pro-inflammatory dysbiosis (microbiome imbalance) in that disorder (Keshavarzian et al., 2015).

Another recent review elucidates additional findings of pertinence to the current discussion (He and Shi, 2017). The combination of prebiotics (dietary fiber that serves as bacterial feedstock, reviewed by Russo, 2016a), and deficient in modern Western diets (Calame et al., 2008; Slavin, 2013) and probiotics may be termed, “synbiotics.” Translocation of bacterial fragments produces “metabolic endotoxemia” from bacterial lipopolysaccharides (LPS). Probiotics may help control PPARγ, “the master regulator of adipogenesis” and TNF-α in inflammation. Additional research supports that prebiotic galacto-oligosaccharides (as from beans) decrease TNF-α, and interleukin production (He and Shi, 2017). GPR41 and GPR43 are orphan receptors for short-chain fatty acids (SCFA) that can increase release of 5-HT and other factors. Additionally, prebiotics change microbiota to reduce adipogenesis and stabilize the gut barrier. Furthermore, CB_2_ levels correlate to *Lactobacillus* concentrations and negatively with potentially pathogenic *Clostridium* species.

Other experiments relate the microbiome to the ECS. A direct effect of *Lactobacillus acidophilus* NCFM strain via oral administration to induce CNR2 (gene encoding the CB_2_ receptor) mRNA expression above that of resting human HT-29 epithelial cells (*p* < 0.01) was demonstrated. An enhancement of morphine antinociceptive effect in rats (*p* < 0.001) was also demonstrated which was inhibited by administration of the CB_2_ antagonist, AM-630 (*p* < 0.001; Rousseaux et al., 2007). Additionally, THC altered the microbiome balance in obese DIO mice affecting the Firmicutes: Bacteroidetes ratio (*p* = 0.021). Furthermore, THC prevented weight gain despite a high-fat diet (Cluny et al., 2015). This explains, perhaps, how the stereotype of the “skinny hippie” is more accurate than that of the lazy, obese “stoner.”

Additional dietary factors include the function of bitter taste receptors (Tepper et al., 2014), present not only on the tongue, but in the gut, and hypothalamus (Herrera Moro Chao et al., 2016), wherein interaction with ECS appetite mechanisms seem to be operative.

Diet is also a key factor in *acne vulgaris*, whose pathophysiology and epidemiology are surprisingly relevant to this discussion. Acne was not observed in Inuit populations living a traditional lifestyle over 30 years, but became common with adoption of a Western diet and lifestyle (Cordain et al., 2002). Similarly, no acne was observed in Papua New Guinea or Paraguay among traditional indigenous peoples. Neither population demonstrated markers of insulin resistance, nor leptin elevations. The author then suggests that in many respects, the epidemiology of acne parallels that of AD. The relationship becomes more salient in light of recent findings (Emery et al., 2017) demonstrating that neuroinflammation is a stimulus to AD development and is triggered by infectious insults. Additionally, AD brains demonstrated 5–10× greater bacterial loads, especially with Actinobacteria, particularly *Propionibacterium acnes*, a gram-positive an aerobic resident of skin, mouth and gut and pathological agent of acne. *P. acnes* has been cultured from AD brains, can grow there, and stimulate alpha synuclein fibrillar formation in PD, amyloid fibrillization in AD, and biofilm formation, which is opposed by cannabinoids, and cannabis terpenoids limonene, alpha-pinene (Soni et al., 2015; Subramenium et al., 2015; Russo and Marcu, 2017).

An additional parallel pertains to the TRPV4 receptor (Zhang et al., 2013). TRPV4 is expressed in cerebral endothelial cells where it mediates Ca^++^ and influx acetylcholine-induced dilation. Cerebral hypoperfusion with impaired vessel dilation is a pathogenetic factor in AD. That function is impaired in a mouse model of AD and is sensitive to oxidative stress from Aβ, which is alleviated by antioxidants. The authors suggested TRPV4 as a target for AD treatment.

Cannabidiol, in addition to its anti-inflammatory and bacteriostatic effects, is a TRPV4 agonist that works as a sebostatic agent in acne (Oláh et al., 2014), while cannabis terpenoids limonene, linalool potently inhibited *P. acnes* and consequent TNF-α production (Kim et al., 2008). Alpha-pinene was also a potent inhibitor of the bacterium (Raman et al., 1995; reviewed by Russo, 2011).

**Figure 2:**
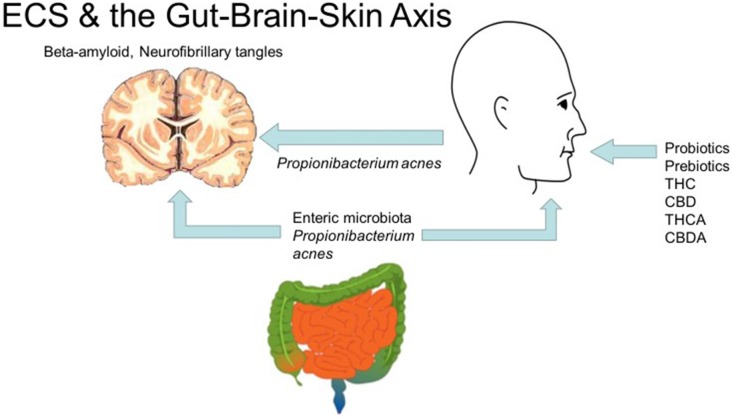
Cannabis, the endocannabinoid system and the gut-brain-skin axis (diagrams of brain, gut by Mikael Hagstrom, face by Mouagip, all public domain).

The importance of these relationships becomes apparent as efforts are made to integrate disparate threads (Bowe and Logan, 2011). Mental health impairment scores in acne patients surprisingly exceed those with epilepsy and diabetes. Oral probiotics regulate inflammatory cytokines in skin. Intestinal microbiota, skin inflammation and psychiatric symptoms are thus intertwined in a “gut-brain-skin axis.” The author posits that acne-induced processes could also affect PD, AD and CTE pathophysiology (Figure 2).

## Future Trends

It is the opinion of many that neurology is facing therapeutic brick walls. The current single target receptor model of pharmacotherapy has not proven universally salutary in the face of complex neurodegenerative diseases. Rather, reconsideration must be given to an older proven model of botanical synergy that may enable polytherapy in single preparations (Russo, 2011; Brodie et al., 2015; Russo and Marcu, 2017; Lewis et al., 2018). Such approaches, combined with nutritional and lifestyle management may make neurology a more preventative and therapeutic specialty, rather than merely diagnostic, and provide better treatment for epilepsy, tumors, AD, PD and TBI/CTE. Suggested strategies include:

- Aerobic activity (Raichlen et al., 2012; Schenkman et al., 2018)
- Education as a lifestyle
- Anti-inflammatory, prebiotic and probiotic diet emphasizing saturated and monounsaturated and omega-3 EFAs, bioflavonoids (berries), fermented foods, protein and minimizing carbohydrates (Fallon and Enig, 1999; Perlmutter and Loberg, 2015)
- Supplementation with cannabis extracts providing THC, CBD, THCA, CBDA, caryophyllene and other select terpenoids (Figure 1; Russo and Marcu, 2017; Lewis et al., 2018).

Legitimate concerns surround the psychoactive sequelae of THC, but as amply demonstrated by the nabiximols RCTs and supported by mitigating effects of cannabidiol and cannabis terpenoids (Russo, 2011; Russo and Marcu, 2017; Lewis et al., 2018; MacCallum and Russo, 2018), cannabis-based drugs portend to provide future safe and effective treatments for heretofore recalcitrant neurological conditions.

## Author Contributions

The author confirms being the sole contributor to this work and has approved it for publication.

## Conflict of Interest Statement

ER is Director of Research and Development for the International Cannabis and Cannabinoids Institute (ICCI), Prague, Czechia.

## Footnotes

^1^https://www.projectcbd.org/

^2^www.gwpharm.com
